# Influence of study model, baseline catalytic concentrations and analytical system on the stability of serum alanine aminotransferase

**DOI:** 10.1515/almed-2020-0021

**Published:** 2020-04-21

**Authors:** Josep Miquel Bauça, Andrea Caballero, Carolina Gómez, Débora Martínez-Espartosa, Isabel García del Pino, Juan José Puente, Maria Antonia Llopis, Itziar Marzana, Marta Segovia, Mercedes Ibarz, Montserrat Ventura, Paloma Salas, Rubén Gómez-Rioja

**Affiliations:** Department of Laboratory Medicine, Hospital Universitari Son Espases Ctra, de Valldemossa, 79, J+1, 07120, Palma, Illes Balears, Spain; Commission on Extraanalytical Quality, SEQC-ML, Spain; Department of Clinical Biochemistry, Clinical Laboratories, Vall d'Hebron University Hospital, Barcelona, Spain; Department of Clinical and Biochemical Analysis, Laboratori Clínic Metropolitana Nord, Germans Trias i Pujol University Hospital, Badalona, Spain; Area Laboratory, Complexo Hospitalario Universitario, A Coruña, Spain; Clinical Biochemistry Laboratory, University Hospital, Zaragoza, Spain; Catalan Health Institute (ICS), Barcelona, Spain; Extranalytical Unit, Laboratories of Cruces University Hospital, Baracaldo, Vizcaya, Spain; Servei d'Anàlisis Clíniques, Hospital Universitari Arnau de Vilanova, IRBLleida, Lleida, Spain; External Quality Assessment Programs, Spanish Society of Laboratory Medicine (SEQC^ML^), Barcelona, Spain; Laboratory Medicine Department, Hospital La Paz-Cantoblanco-Carlos III, Spain

**Keywords:** alanine aminotransferase (ALT), enzyme, preanalytical phase, stability, storage

## Abstract

**Objectives:**

The stability of the analytes most commonly used in routine clinical practice has been the subject of intensive research, with varying and even conflicting results. Such is the case of alanine aminotransferase (ALT). The purpose of this study was to determine the stability of serum ALT according to different variables.

**Methods:**

A multicentric study was conducted in eight laboratories using serum samples with known initial catalytic concentrations of ALT within four different ranges, namely: <50 U/L (<0.83 μkat/L), 50–200 U/L (0.83–3.33 μkat/L), 200–400 U/L (3.33–6.67 μkat/L) and >400 U/L (>6.67 μkat/L). Samples were stored for seven days at two different temperatures using four experimental models and four laboratory analytical platforms. The respective stability equations were calculated by linear regression. A multivariate model was used to assess the influence of different variables.

**Results:**

Catalytic concentrations of ALT decreased gradually over time. Temperature (−4%/day at room temperature vs. −1%/day under refrigeration) and the analytical platform had a significant impact, with Architect (Abbott) showing the greatest instability. Initial catalytic concentrations of ALT only had a slight impact on stability, whereas the experimental model had no impact at all.

**Conclusions:**

The constant decrease in serum ALT is reduced when refrigerated. Scarcely studied variables were found to have a significant impact on ALT stability. This observation, added to a considerable inter-individual variability, makes larger studies necessary for the definition of stability equations.

## Introduction

For laboratory tests to be of clinical usefulness, the composition of the samples tested *in vitro* should reflect their real composition *in vivo*. To such purpose, constituent concentrations must remain stable during the preanalytical period. The stability of biochemical analytes is defined as the ability of a sample to retain the initial value of its constituents within specified limits over a period of time under specified storage conditions [1, 2]. The stability of the biological magnitudes most frequently measured in serum and plasma under specific conditions in clinical laboratories has been analyzed in numerous studies, with varying and often conflicting results. The inconsistency of results may arise from the lack of standardized experimental protocols, guidelines or validated recommendations about analyte stability testing, and the absence of standard maximum permissible instability specifications [3–5]. In 2006, the Spanish Society of Laboratory Medicine (SEQC^ML^) discussed this problem and developed a stability testing protocol. In 2019, a new version of the protocol was published to include a preliminary study and a complete stability test to define a stability equation [1].

Alanine aminotransferase (ALT, EC.2.6.1.2) is an enzyme whose stability in the short term (seven days) has been intensively studied, with inconsistent results, which hinders the development of a consensus stability equation [3]. Some studies show significant instability of ALT in whole blood [6–8] or in serum stored at 4 °C [9–11]. Other studies reveal increased catalytic concentrations both in whole blood and serum/plasma [12, 13]. In contrast, most authors report a decrease of catalytic concentrations at room temperature (22–25 °C) [9, 14], under refrigeration [15], and at −20 °C [9, 14, 16].

In light of the inconsistency of results, a multicentric study was conducted in eight hospitals to assess the stability of ALT using four experimental models and four analytical platforms. As variables with a potential effect on stability, we included time, temperature and the initial catalytic concentration of the enzyme.

## Materials and methods

A multicentric study was performed in eight hospitals in Spain. Serum samples with known initial catalytic concentrations of ALT were stored for seven days at two temperatures (room temperature and refrigerated) and analyzed using four experimental models:

Model 1: approximately 20 serum samples were selected (five samples with ALT > 400 U/L (>6.67 μkat/L), five samples with 200 < ALT < 400 U/L (3.33 < ALT < 6.67 μkat/L), five samples with 50 < ALT < 200 U/L (0.83 < ALT < 3.33 μkat/L), and five samples with ALT < 50 U/L (<0.83 μkat/L). Samples were divided into ten aliquots that were transferred to Eppendorf tubes. Five aliquots of each sample were stored at room temperature, whereas the other five were stored refrigerated. Tubes were capped and protected from light without shaking. An aliquot of each sample was frozen at −70 °C at intervals of 0, 1, 2, 3, and 7 days.

On day 8, all aliquots were thawed and analyzed in the same run. To minimize contamination, the samples with the lowest catalytic concentrations of ALT were first analyzed.

Model 2: approximately 40 serum samples were selected: ten samples with ALT > 400 U/L (>6.67 μkat/L), ten samples with 200 < ALT < 400 U/L (3.33 < ALT < 6.67 μkat/L), ten samples with 50 < ALT < 200 U/L (0.83 < ALT < 3.33 μkat/L), and ten samples with ALT < 50 U/L (<0.83 μkat/L). After the first analysis, primary tubes were sealed immediately. Half of the aliquots were stored refrigerated and the other half were stored at room temperature protected from light without shaking. Aliquots were transferred into the tubes and analyzed at intervals of 0 (repeat initial value), 1, 2, 3, and 7 days. In each case, the remainder of the sample was stored in the tube and immediately sealed.

To minimize contamination, the samples with the lowest catalytic concentrations of ALT were first analyzed.

Model 3: approximately 200 serum samples were selected: 50 samples with ALT > 400 U/L (>6.67 μkat/L), 50 samples with 200 < ALT < 400 U/L (3.33 < ALT < 6.67 μkat/L), 50 samples with 50 < ALT < 200 U/L (0.83 < ALT<3.33 μkat/L), and 50 samples with ALT < 50 U/L (<0.83 μkat/L). Immediately after the first result was obtained, half of the primary tubes were stored refrigerated, whereas the other half were stored at room temperature. All tubes were sealed and stored protected from light without shaking.

From the day of collection, each day 14 tubes stored at room temperature and another 14 refrigerated were reanalyzed in the laboratory at normal working hours.

Model 4: approximately 20 serum samples were selected: five samples with ALT > 400 U/L (>6.67 μkat/L), five samples with 200 < ALT < 400 U/L (3.33 < ALT < 6.67 μkat/L), five samples with 50 < ALT < 200 U/L (0.83 < ALT < 3.33 μkat/L), and five samples with ALT < 50 U/L (<0.83 μkat/L). Within the first 2 h after centrifugation, four pools were prepared (one pool for each ALT catalytic concentration range). Each pool was divided into ten aliquots, which were transferred into Eppendorf tubes. Five aliquots were stored at room temperature, whereas the other five were stored refrigerated. All tubes were sealed and stored protected from light without shaking. An aliquot of each sample was frozen at −70 °C at intervals of 0, 1, 2, 3, and 7 days.

On day 8, all aliquots were thawed simultaneously for analysis in quintuplicate in the same run.

Each model was tested in different laboratories using different analytical platforms (Table 1). The incubation temperature at room temperature was set at 20–24 and 4–8 °C for refrigeration.

**Table 1:** Characteristics of the analytical platforms used in the different experimental models.

Experimental Model	Analytical platform	Method (ALT assay)	Number of samples tested
1	AU (Beckman Coulter)	NADH with PyP	156
	Architect c16000 (Abbott)	NADH without PyP	160
	Advia 2400 (Siemens)	NADH without PyP	160
2	AU (Beckman Coulter)	NADH with PyP	160
	Advia 2400 (Siemens)	NADH without PyP	96
	Cobas c702 (Roche)	NADH without PyP	194
3	Advia 2400 (Siemens)	NADH without PyP	242
4	Cobas c702 (Roche)	NADH without PyP	80
	Architect c16000 (Abbott)	NADH without PyP	160

PyP, pyridoxal-5′-phosphate; NADH, nicotinamide adenine dinucleotide.

Tubes were capped or sealed and stored protected from light without shaking to avoid the effects of other factors of influence. The quality of results was assured by the use of laboratory internal quality control measures. Hemolyzed or lipemic samples were excluded.

### Statistical analysis

Percent deviation (PD) from the initial value was used as the basic statistic (initial catalytic concentration − final catalytic concentration)/initial catalytic concentration × 100). Following SEQC^ML^ guidelines, we determined the stability equation showing the relationship between PD variations over time by linear regression using the least squares method [1]. The model was forced to include the coordinate origin, as at time 0 loss of stability must be 0. A confidence interval of 95% was calculated for the slope. Visual inspection of results suggested a first-order linear fit, which is easier to interpret. Therefore, all models followed:

PD=β×time[days]

(*β* = daily percent deviation from initial ALT catalytic concentration).

The level of significance for comparison tests (p) and the goodness-of-fit (Pearson's r) of each stability equation are provided.

A stepwise multiple linear regression was performed to test the individual effect of each variable (temperature, initial ALT catalytic concentration, experimental model and analytical platform). An *α* error of 0.05 was set for inclusion of variables.

In the case of analytical platform or initial catalytic concentration, univariate analysis of variance (ANOVA) was performed to assess PD of results on day 7. The individual difference between categories was determined using the least significant difference test (LSD).

Informed consent from patients was not required as waste samples were used in this study. All data were previously anonymized.

## Results

Table 1 shows the analytical features of each experimental model and the number of samples tested.

In general terms, temperature demonstrates to exert clear effects on the stability of ALT in serum, with a decrease of 4.77%/day at room temperature, as compared to a decrease of 0.94%/day under refrigeration. Table 2 describes the stability equations obtained by each of the participating laboratories for samples stored at room temperature and refrigerated.

**Table 2:** Variations in catalytic concentrations of ALT over time. Stability equations.

Model	Platform	Refrigerated (n = 704)				At room temperature (n = 704)			
		p-Value	r	*β*	95%CI *β*	p-Value	r	*β*	IC 95% *β*
1	Advia	0.001	0.661	−0.902	between −1.393 and −0.410	<0.001	0.947	−3.87	between −4.504 and −3.248
1	Architect	<0.001	0.500	−1.117	between −1.550 and −0.683	<0.001	0.903	−8.742	between −9.673 and −7.811
1	AU	<0.001	0.677	.0.695	between −0.865 and −0.525	0.001	0.679	−3.384	between −5.017 and −1.590
2	AU	<0.001	0.752	−1.251	between −1.496 and −1.005	<0.001	0.942	−5.199	between −5.613 and −4.785
2	Cobas	<0.001	0.870	−1.607	between −1.796 and −1.417	<0.001	0.908	−5.824	between −6.353 and −5.294
2	Advia	<0.001	0.626	−1.321	between −1.783 and −0.858	<0.001	0.829	−5.048	between −6.096 and −4.000
3	Advia	<0.001	0.345	−0.605	between −0.902 and −0.307	<0.001	0.822	−2.189	between −2.463 and −1.915
4	Cobas	0.506	0.107	0.110	between −0.222 and 0.442	<0.001	0.939	−2.001	between −2.250 and −1.752
4	Architect	0.001	0.720	−1.025	between −1.569 and −0.481	<0.001	0.855	−5.609	between −7.484 and −3.733
Total		<0.001	0.564	−0.941	between −1.051 and −0.831	<0.001	0.832	−4.772	between −5.025 and −4.518

p, level of significance; r, goodness of fit (Pearon's r); *β*, daily percent deviation from ALT catalytic concentration.

**Figure 1:**
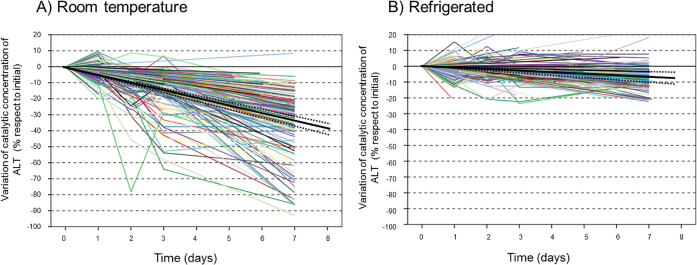
Effect of temperature and experimental model. Individual data and linear regression (black solid line) with a 95% CI for the slope (dotted lines). (A) Tests at room temperature. (B) Test under refrigeration.

Inter-individual variability in loss of stability is significantly higher in the samples stored at room temperature (Figure 1). The graphs suggest a general model of linear reduction of catalytic concentrations over time for all experimental models and analytical platforms. The improvement of the R^2^ coefficient with quadratic or cubic fitting was not significant either at room temperature (0.545 for linear fitting vs. 0.547 for quadratic and 0.558 for cubic) or refrigerated (0.165 vs. 0.166 and 0.189).

**Figure 2:**
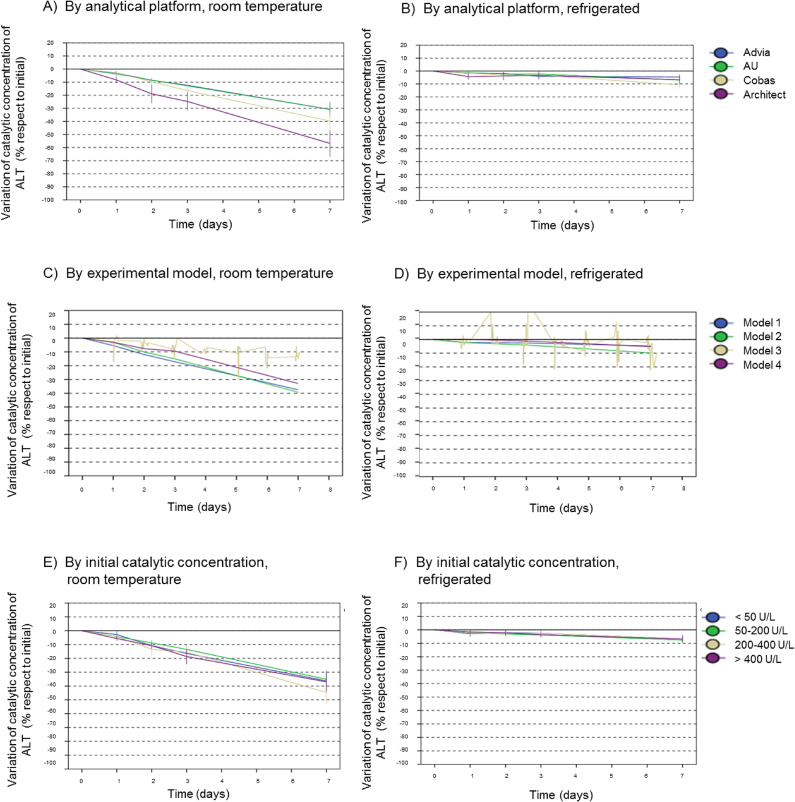
Variations in catalytic concentrations of ALT over time as a function of the different variables. (A, B) Analytical platform, (C, D) experimental model, (E, F) initial catalytica concentration. Represented as line plot adding the means for each time point with the 95% confidence interval for the mean.

Figure 2 displays variations in ALT catalytic concentrations over time based on the experimental model (2A), the initial ALT catalytic concentration (2B), and the analytical platform (2C).

Stepwise multiple linear regression analysis was performed including the five variables (by order: time, temperature, analytical platform, experimental model and, initial catalytic concentration). An increase of 0.05 in the probability of F was considered significant. All variables showed a significant effect on variations in the catalytic concentrations of ALT, except for experimental model. The explanatory power of the model increased from R^2^ = 0.448 when time was included as the only predictor, to R^2^ = 0.483 when temperature was included, to R^2^ = 0.594 when the method was included, and R^2^ = 0.602 when initial catalytic concentration was incorporated, being the latter the variable with the lowest weight.

Differences between mean PD values at day 7 for the four analytical platforms were assessed by an ANOVA test including all results (both for samples stored at room temperature and refrigerated). A significant difference was found between platforms (ANOVA p < 0.01) with a more substantial loss of stability in Architect-Abbott (−31.8%), followed by Cobas-Roche (−26.3%), AU-Beckman (−18.4%), and Advia-Siemens (−14.5%). The LSD test revealed significant differences between Abbott or Cobas and AU or Advia. Differences were also observed between study models (ANOVA p < 0.01), primarily between model 3 (−8.8%) and the other models (−20.7% for model 1, −24.8% for model 2, and −19% for model 4).

The influence of initial catalytic concentration detected by multiple regression was not seen in day 7 results (ANOVA p = 0.430), which corresponds to the small difference seen in the graph.

## Discussion

This multicentric study was performed to assess the influence of different variables on variations in the catalytic concentrations of ALT over time. All experimental designs showed a reduction of catalytic concentrations over time, which is consistent with the results of some previous studies [3, 17, 18], although our results also contrast with other authors who did not find any variation [19–21] or report an increase in catalytic concentrations of this enzyme [22].

According to our results, temperature proved to be a significant factor of influence on stability, with stability being five-fold higher in samples stored refrigerated, as compared to storage at room temperature. The greatest loss of stability occurs at room temperature, which is in line with the literature [22, 23].

To the best of our knowledge, this is the first study to assess the influence of variables that had not been considered in previous studies on ALT stability. These variables include initial catalytic concentration, analytical platform and the experimental model [7, 9, 11].

Catalytic concentrations (especially high concentrations) have not been considered in previous studies on ALT stability. A limitation of previous studies is that they were based on low catalytic concentrations or on concentrations within the reference range [8, 10, 13, 14, 16]. Hence, in many studies, analytical variability could have masked variations caused by the loss of stability. In our study, we considered catalytic concentration groups over the analytical range, which is of greater clinical usefulness, from <50 U/L (0.83 μkat/L) to >400 U/L (3.33 μkat/L). Multivariate analysis shows a weak influence of initial catalytic concentration, with samples with high values showing the greatest instability. Notably, this effect does not seem to be maintained until day 7, which suggests that this effect could stem from a greater imprecision of ALT quantification methods in samples with a low catalytic concentration.

Four analytical platforms were included in multivariate analysis. Loss of stability was more relevant when Architect or Cobas were used, as compared to Advia or AU. The greatest instability was observed in Architect. This phenomenon may be explained by the differential composition of the reagents used by the analyzers and the addition of pyridoxal-5′-phosphate (PyP) as a coenzyme, which is necessary to improve the stability of the method. The methods where PyP is not added depend on the presence of endogenous PyP in the sample. PyP degradation with exposure to light is widely known, as is the need to refrigerate samples. Further studies are warranted to investigate the potential relationship between PyP and catalytic concentrations of ALT.

In our study, stability was assessed in different experimental models. In their recent guidelines for analyte stability testing, SEQC^ML^ recommended the use of an equation or profile instead of a single stability cut-off value [1]. Based on a stability equation, laboratories can set their cut-off points according to the established specifications.

For results to be reliable, the use of an appropriate experimental model is of great relevance. In our study, the models proposed have advantages and disadvantages in terms of viability of the model and interpretation of results. For example, models 1 and 4 are based on the assumption that no loss of stability occurs when the sample is stored at −70 °C. Therefore, model 2 is recommended for samples that cannot be frozen or when catalytic concentrations become unstable with freezing. Nevertheless, control materials from the same batch should have been used to assess potential biases.

Model 1 is very similar to that described in a recently published stability testing protocol [1]. However, a limitation of models 1, 2, and 3 is that determinations were performed once and outliers may have not been detected. Analysis in quintuplicate of model 4 enables to discard outliers and reduces the imprecision of results. The main limitation of this model is the small number of samples used (four pools). In spite of multiple differences among the four experimental models, the multiple regression model only showed differences in day-7 results. A potential confounding factor is the fact that model 3 is the only model were determinations were not performed at specific time points, and this model was only tested in a laboratory due to its technical complexity. The platform used to test model 3 (Advia Siemens), together with AU Beckham was the one with the lowest loss of stability, which may explain differences with other models.

The main limitations of this study are the small number of analytical platforms tested and the low number of repetitions in some of them. Moreover, the number of samples included in some of the experimental models was low, which hinders drawing robust conclusions. Investigating the influence of other factors found in routine laboratory practice such as exposure to air or light on ALT or PyP concentrations is a worthwhile avenue for future research.

Individual results grouped by factor of influence revealed significant inter-individual variability in terms of ALT stability. This finding demonstrates the limitations of and difficulty in establishing a fixed and single stability cut-off value for a biochemical magnitude for the general population under specific conditions. Thus, the samples of some patients lose stability more rapidly (those with a sharper slope in Figure 1) and their results would be considered to be “within the stability period”, when their sample actually is no longer stable. The opposite occurs in individuals with very stable catalytic concentrations of ALT over time, whose samples can be stored for longer. This effect could be partially explained by the presence of thermosensitive isoenzymes, as documented in lactate dehydrogenase [23].

It could be hypothesized that the same phenomenon occurs in other analytes of clinical interest. Thus, stability cannot be determined at a single time point or using a single equation. However, this is the best tool currently available in clinical laboratories to test stability.

This study brings out the necessity of assessing the influence of a variety of factors in the stability of biochemical magnitudes, some of which with a weak effect in the case of ALT (initial catalytic concentrations or experimental model), whereas other factors may have a significant impact (storage temperature or analytical platform). Graphical representations showing the stability of all samples allow a visual inspection of inter-individual variability in the loss of stability. Defining a stability line can be useful for each laboratory to set their stability limits according to their specifications.
